# A Territorial's "Internment" in an Isolation Hospital

**Published:** 1915-04-03

**Authors:** 


### FROM THE PATIENT'S POINT OF VIEW.

(Contributions to this Section are Invited.)

### A Territorial's "Internment" in an Isolation Hospital.

I JOINED the Territorials at the beginning of September last, and up to the end of November was engaged in the usual drills, and more especially in signalling. I then applied for and obtained leave to visit relations for a week-end. On Saturday, November 28, after a route march of fourteen miles, I departed for my destination, arriving there about 9.30 P.M. After a rotten night and a ditto morning I was shepherded by my aunt to the doctor, who examined me and informed me that I was possessed of a fine rash, a temperature of 104°, and scarlet fever. I was told to go back to the house I was staying at and wait until called for. I must explain that patients are usually fetched in the ambulance to the hospital, but I had walked down to the doctor, and he saw no reason, it being Sunday, why I should not walk there. After twenty minutes the sanitary inspector appeared and piloted me to the isolation hospital, which was a corrugated-iron building with matchboard interior.

On entering, my temperature was taken, and the nails of my fingers and toes cut; next I underwent the order of the bath, and was quickly ensconced between the sheets. As it was my first introduction to a hospital I was wondering somewhat as to the treatment I should experience. However, I was put thoroughly at ease by the matron, and commenced to enjoy the experience very soon.

The fever had a firm hold on me, so I was informed, for about a fortnight, but thanks to the attentions of the matron I did not experience any of the complications of scarlet fever. My head for the first few days was like a lump of lead, and it was somewhat of a struggle to sit up for my two-hourly gargle and feeds, as the effort made the muscles of the neck and back ache. After a week's diet of milk I was allowed to commence gradually on food of a more substantial character. During this week I was not allowed to shave, but two days after I started solid food I was told I could have one—I whistled when I saw my face in the glass; it looked something like a coconut mat. A few more days passed, and permission was obtained from the doctor for me to rise, at which I was much pleased.

As I was the only adult the matron christened me "Baby," and great was the enjoyment of the children when on visiting days (Wednesday and Sunday afternoons from 3 to 5 P.M.) matron entered the ward and said, "Baby, someone to see you."

The following was the order and time of the meals, etc.: 6.40 A.M., patients washed; 7.0, breakfast; 9.0, patients' feet soaked and scraped; 10.0, drink of milk; 12.30 P.M., dinner; 4.30 P.M., tea; 8 P.M., drink of milk, lights lowered, and tucked up for the night; matron coming in as occasion demanded.

At first I was rather wobbly about the knees, but after three or four days the wobbliness passed away. My time was mostly occupied in reading, but a week before Christmas instructions were given that I was to commence to decorate the wards, both wards to be exactly the same as each other—I was not in any way to make the boys' ward more elegant than the girls'. The chief ornamentation consisted of laurel leaves threaded on cotton, and then paper festoons were also hung about until the wards looked quite Christmassy. When Christmas morning dawned we all began to empty our stockings. In the evening the Christmas-tree—it was constructed with branches of box—was dismantled and the presents packed away until the recipients were discharged. On the Monday after Christmas the wards were cleared of decorations. During the whole of the time patients were being discharged and fresh arrivals came to take their places, the average during my stay was about twelve.

### AN "AMUSING" EXPERIENCE.

After Christmas the most amusing of my experiences began to happen. After soaking my feet in hot water for some time I was told to get on the table and lie on my stomach, and then for an hour the matron scraped and dug with an old knife at my feet to get the hard skin off. The unoccupied foot somehow would keep on twitching and waving in the air, and I was asked if I was signalling to anybody. This recreation lasted the best part of three weeks.

During the last three weeks of my stay I began to get so "plump" that my belt became uncomfortably tight. I was assured it was only my fancy, but when I did get out and met my chums I was greeted with the epithet "Porky."

When the day of my discharge drew nigh my wearing apparel and articles appertaining to the toilet were taken away and fumigated, and the last day I was attired in a waistcoat and trousers belonging to the caretaker, in which I could have put two of my size. After my disinfecting bath, I dined in the matron's room; the hospital consisted of only two wards, kitchen, and the matron's room. Finally, I took my departure from the hospital about 3.30 P.M., after spending on the whole a most enjoyable holiday.

